# A simple tool for neuroimaging data sharing

**DOI:** 10.3389/fninf.2014.00052

**Published:** 2014-05-21

**Authors:** Christian Haselgrove, Jean-Baptiste Poline, David N. Kennedy

**Affiliations:** University of Massachusetts Medical SchoolWorcester, MA, USA

**Keywords:** neuroinformatics, neuroimaging, quality assessment, data processing, data archiving

## Abstract

Data sharing is becoming increasingly common, but despite encouragement and facilitation by funding agencies, journals, and some research efforts, most neuroimaging data acquired today is still not shared due to political, financial, social, and technical barriers to sharing data that remain. In particular, technical solutions are few for researchers that are not a part of larger efforts with dedicated sharing infrastructures, and social barriers such as the time commitment required to share can keep data from becoming publicly available. We present a system for sharing neuroimaging data, designed to be simple to use and to provide benefit to the data provider. The system consists of a server at the International Neuroinformatics Coordinating Facility (INCF) and user tools for uploading data to the server. The primary design principle for the user tools is ease of use: the user identifies a directory containing Digital Imaging and Communications in Medicine (DICOM) data, provides their INCF Portal authentication, and provides identifiers for the subject and imaging session. The user tool anonymizes the data and sends it to the server. The server then runs quality control routines on the data, and the data and the quality control reports are made public. The user retains control of the data and may change the sharing policy as they need. The result is that in a few minutes of the user’s time, DICOM data can be anonymized and made publicly available, and an initial quality control assessment can be performed on the data. The system is currently functional, and user tools and access to the public image database are available at http://xnat.incf.org/.

## INTRODUCTION

Data sharing is becoming increasingly common (Biswal et al., 2010; Di Martino et al., 2013), but despite encouragement and facilitation by funding agencies, journals, and some labs and larger research efforts^1^ (Hall et al., 2012; Prior et al., 2013), there remain political, financial, social, and technical barriers to sharing data (Poline et al., 2012). Excuses such as “it’s too hard” and “it takes too long” are all too common, and there is anxiety about subject protection and control of data (De Schutter, 2010). And unless one is part of a large project with dedicated sharing infrastructure, there is also a lack of open technical infrastructure and public and free archive space.

There are some central, open databases for image data sharing such as The Cancer Imaging Archive^2^ and the National Database for Autism Research^3^, but these are domain-specific, and contributing data requires a substantial investment of time to handle both bureaucratic and technical aspects of contributing data. On the other end of the spectrum are image databases that can be installed locally, such as COINS^4^ (Scott et al., 2011), the Human Imaging Database^5^ (Ozyurt et al., 2010), LORIS^6^ (Das et al., 2012), NIDB^7^ (Book et al., 2013), and XNAT^8^ (Marcus et al., 2007). Using any of these to share image data requires an investment in hardware as well as initial and ongoing technical support. With the exception of XNAT Central, none of these provide a public, open instance that anyone can use to share their data.

Given an open repository such as XNAT Central, other issues come into play. The actual mechanics of uploading data must then be addressed. There are tools available to facilitate data upload, but these often require somewhat involved installation, and most are then general in scope, with many options that must be understood. XNAT Desktop^9^ and DicomBrowser^10^, for instance, allow a user to manage local data and send it to XNAT Central, but the flexibility in anonymization options and subject identifier customization mean that there is a learning curve to using these tools effectively. Moreover, they often don’t capture the relevant metadata simply and efficiently.

We have created a system for data sharing that attempts to address many of these issues. We set up a public, open image repository within an international organization that can host and manage imaging data, and have created user tools that make data upload to this server trivial. The user software is designed to be easy to install, and once installed, data upload is initiated by a simple drag and drop. The user is then walked through the few steps necessary to anonymize and upload the data in a way that control of the data on the repository is retained. On receipt of the data, the repository also runs quality assessment (QA) routines on the data as a service to the user and as additional motivation to share. In the near future, this should also provide the imaging community with a useful resource for quality checking. This report describes the design and implementation of this system and initial results of its testing and validation.

## METHODS

### OVERVIEW

**FIGURE 1:**
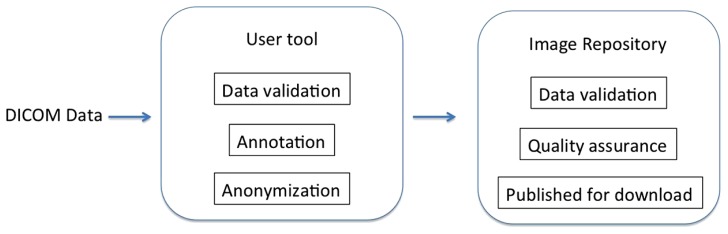
**System overview.** Users are walked through data preparation using the user tool, after which the data is sent to the image repository for further processing and publishing.

The system has two components: the image repository and the user tools. The repository is an XNAT installation, and while some XNAT customizations were necessary, most of the innovation lies with the user tools. An overview of the design of the system can be found in **Figure 1**. Since the overarching goal of this system is to make data sharing simple, we describe the components of the system in the order they are encountered by the data as it moves from a local disk to the server. The ultimate effect is that given a few minutes of a researcher’s attention, data is anonymized, archived, and shared, and the researcher gets feedback on the quality of the data.

The server itself can be found at http://xnat.incf.org, and the user tools can be downloaded from this location as well. Source code for the user tools and custom code for the repository can be found on GitHub at http://github.com/incf/one_click.

### USER UPLOAD TOOL

The driving design principle for the user tool is ease of use. Our goal is to remove the barriers to data sharing, and the more difficult it is to install or successfully run any tool, the more likely it is that the user will give up. We provide two user tools, a command line script and a graphical user interface (GUI). The two options provide the same functionality, but in different ways: the command line script is useful for users comfortable at the command line, while the GUI uploader is useful for users accustomed to a more interactive experience. The only requirement for these tools is that data is prepared in a certain well-defined way before being sent to the archive (see below).

The current user tools are written in Python, released under the BSD license, and can be installed on Linux or Mac OS machines. Dependencies are pydicom^11^, httplib2^12^, and DCMTK^13^. The user tools can be downloaded directly from the International Neuroinformatics Coordinating Facility (INCF) web site^14^. The command line tool requires manual installation of the dependencies, although it is packaged and released through NeuroDebian^15^ (Halchenko and Hanke, 2012) which simplifies installation and dependency handling on Debian systems. The Linux GUI tool also requires PyQt^16^. All of the dependencies are bundled for the Mac OS GUI.

The custom code for the archive server is also made available on line via GitHub^17^ and released under the BSD license. Although we plan to support the ability to push to alternate archives, focus so far has been on the user tools and user experience, with one archive sufficient for testing. Similar to a new user tool, a new archive for this system would only have to conform to certain well-defined specifications, such as being able to handle data prepared as described below.

#### Data selection, validation, and annotation

**FIGURE 2:**
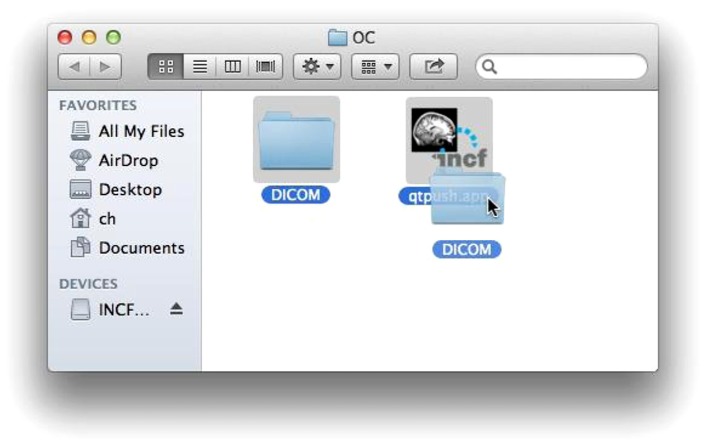
**Selecting data for upload.** Dragging and dropping the DICOM folder to the uploader application initiates the process using the GUI user tool.

The first step is selecting the Digital Imaging and Communications in Medicine (DICOM) data to share. This can be invocation of the command line script that takes the containing directories of the data as arguments or dragging and dropping a folder containing data onto the GUI tool (**Figure 2**). The selected data is then validated and sorted into subjects and imaging sessions: the user tool scans the specified directories for DICOM data using pydicom and groups the data by subject (by the DICOM Patient ID field) and imaging session (by Study Instance UID).

**FIGURE 3:**
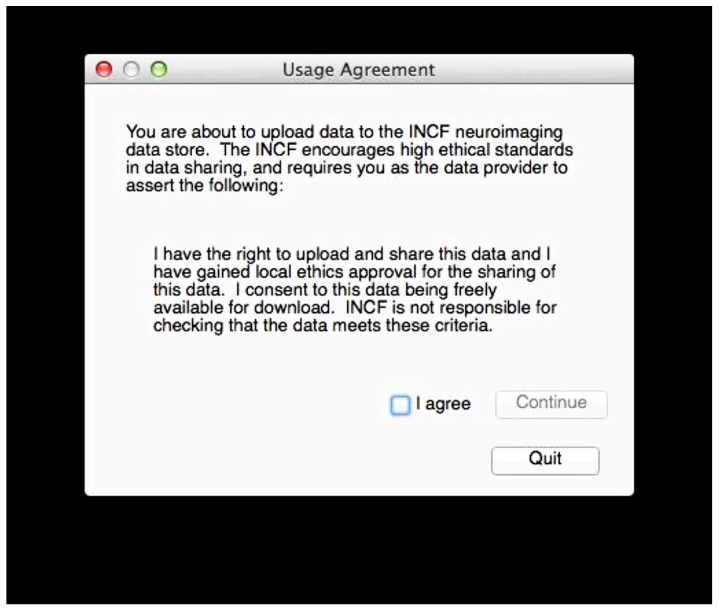
**Upload agreement.** After verifying that DICOM data is available in the selected folder and before further action, the user must consent to this agreement.

**FIGURE 4:**
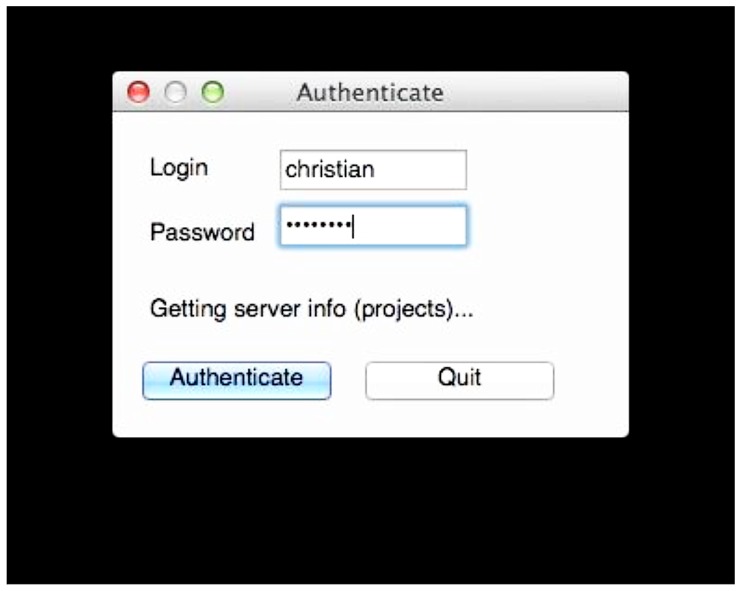
**User authentication.** The uploaded data is tagged with the user name so the user retains control of the data. Requiring the password at this stage allows the tool to query the archive for existing data so conflicts can be avoided when labeling the data. Here, the user tool is querying the archive for existing projects.

If valid data is found, the user is asked to consent to a simple usage agreement before proceeding (**Figure 3**). This agreement is intentionally broad and simple; waiting to implement this upload system until all of the legal aspects of sharing have been perfected is a recipe for failure. The user is then prompted for a user name and password that identify the user on the INCF portal^18^. The user name allows the archive to assign the data to the user so they retain control of the data, and links to the e-mail address to which reports are sent. Since the archive shares the users and passwords of the INCF portal, the password allows the user tool to query the archive for existing data under the user’s control to avoid collisions of new subject or session identifiers (**Figure 4**). This all takes a few short minutes of the user’s time and attention.

**FIGURE 5:**
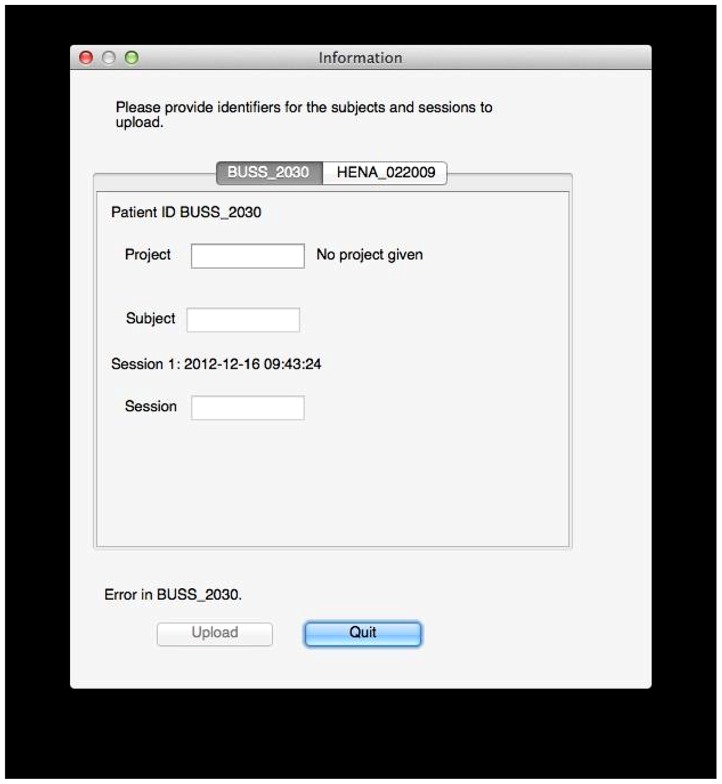
**Data labeling.** In this example, the user tool found data for two subjects, BUSS_2030 and HENA_022009. The user now selects a project and specifies public subject and session identifiers for the data on the archive. Validation is done on the fly: here, an error exists because no project is given, but the tool will also inform the user if he does not have sufficient permissions to upload to the specified project, if the session already exists, if identifiers use invalid characters, and so on.

There is some coordination with the archive required at this stage. The archive server is running XNAT, which provides a set of REST^19^ services that allow these queries. XNAT structures data hierarchically into projects, subjects, and sessions. Permissions are handled at the project level: access to subjects and sessions depend solely on the level of access permitted to the containing project. The user tool prompts the user for a project for each subject in the selected data, and since the tool has queried the archive, the tool can verify that the user is specifying a project to which he has access or a new project that can be created. Similarly, the user tool will prompt the user for valid subject and session identifiers that do not conflict with those already in the archive (**Figure 5**).

#### Anonymization and upload

The data is anonymized locally before it leaves the user’s computer and is then sent to the archive. At this stage, all necessary information has been collected from the user, and the data must be prepared and sent to the archive. One benefit of using DICOM data in our initial test case is that the DICOM standard includes a network communication protocol for transferring data, a protocol which XNAT handles natively on the receiving end. But the user tool must first anonymize the data so no identifiable information leaves the user’s machine and then annotate the data with the user information and the specified data identifiers. Depending on the amount of data and the quality of the network connection, this may take an hour or more, but it does not require the user’s attention.

Anonymization is a challenge because of the various levels and interpretation of anonymization that can be applied. DICOM defines concepts such as patient name and study date that it stores in fields, and there are several different conflicting DICOM anonymization schemes that specify what information should be protected (meaning, in our case, removed from the data). We can illustrate this challenge by examining three different examples of existing anonymization protocols: DICOM Supplement 55^20^ (developed primarily with clinical uses in mind), the National Cancer Institute deidentification profile^21^, and the default deidentification profile provided by XNAT’s DICOM Browser^22^. All agree that the Patient’s Name field should be protected, but only one specifies protecting Study Date, another protects Patient’s Address, one pair protects Patient’s Age, and another pair protects Institution Name, and so on in every combination. Clearly, no consensus is to be found: the level of anonymization depends on the application context and the specifics of the data. In addition, the DICOM specification defines fields that must be present in valid data sets^23^, and programs at both the sending and receiving ends of the network transfer have their own quirks regarding what fields they require to be present.

Rather than trying to definitively solve this problem, we decided to choose a set of protected fields that are removed or replaced (guided by existing anonymization profiles), making sure that the network tools on either end would function with our anonymized data. **Table 1** shows the protected fields that are currently removed from the data before it is sent to the archive.

**Table 1:** DICOM fields for anonymization.

Tag	Name
(0008, 0050)	Accession number
(0008, 0080)	Institution name
(0008, 0090)	Referring physician’s name
(0008, 0096)	Referring physician identification
(0008, 1048)	Physician(s) of record
(0008, 1049)	Physician(s) of record identification
(0008, 1050)	Performing physicians’ name
(0008, 1052)	Performing physician identification
(0008, 1060)	Name of physician(s) reading study
(0008, 1062)	Physician(s) reading study identification
(0010, 0030)	Patient’s birth date
(0010, 0050)	Patient’s insurance plan code
(0010, 0101)	Patient’s primary language code
(0010, 1000)	Other patient IDs
(0010, 1001)	Other patient names
(0010, 1002)	Other patient IDs
(0010, 1005)	Patient’s birth name
(0010, 1010)	Patient’s age
(0010, 1040)	Patient’s address
(0010, 1060)	Patient’s mother’s birth name

*The current user tools clear or remove values for these fields, and data that arrives at the archive with any of these fields set is rejected.*

The INCF user name and the project, subject, and session identifiers specified by the user are stored in the Study Comments field, which is replaced or created as needed.

This anonymized and annotated DICOM data is then pushed to the archive using the DICOM network transport protocol by storescu from the DCMTK package. Similar to the Python dependencies described above, this can be installed separately, but NeuroDebian handles its installation on Linux and it is bundled with the Mac OS GUI tool.

### IMAGE REPOSITORY

The repository itself is located at and hosted by the International Neuroinformatics Coordinating Facility (INCF). The server itself is a Linux virtual machine with two 2.4 GHz processors and a total of 4 GB memory. The image repository is a customized installation of XNAT 1.5.4.

#### Data validation and archiving

Data is validated on arrival at the archive and then archived. The server itself does not have the processing power or memory for intensive parallel analysis, so launching this computationally intensive processing immediately when data arrives could easily overload the system if a lot of data arrives at once. This step is therefore queued and run using the arc-queue tools^24^.

The validation processing starts with an anonymization check, and if the data does not conform to the anonymization profile described above (i.e., if any of the protected fields are found in the data), the data is removed from the archive and the user is notified by e-mail. The content of the Study Comments field is then validated, checking for a valid user and for project, subject, and session identifiers. If the project exists, user permissions are also checked. If everything is in order, archiving begins.

**FIGURE 6:**
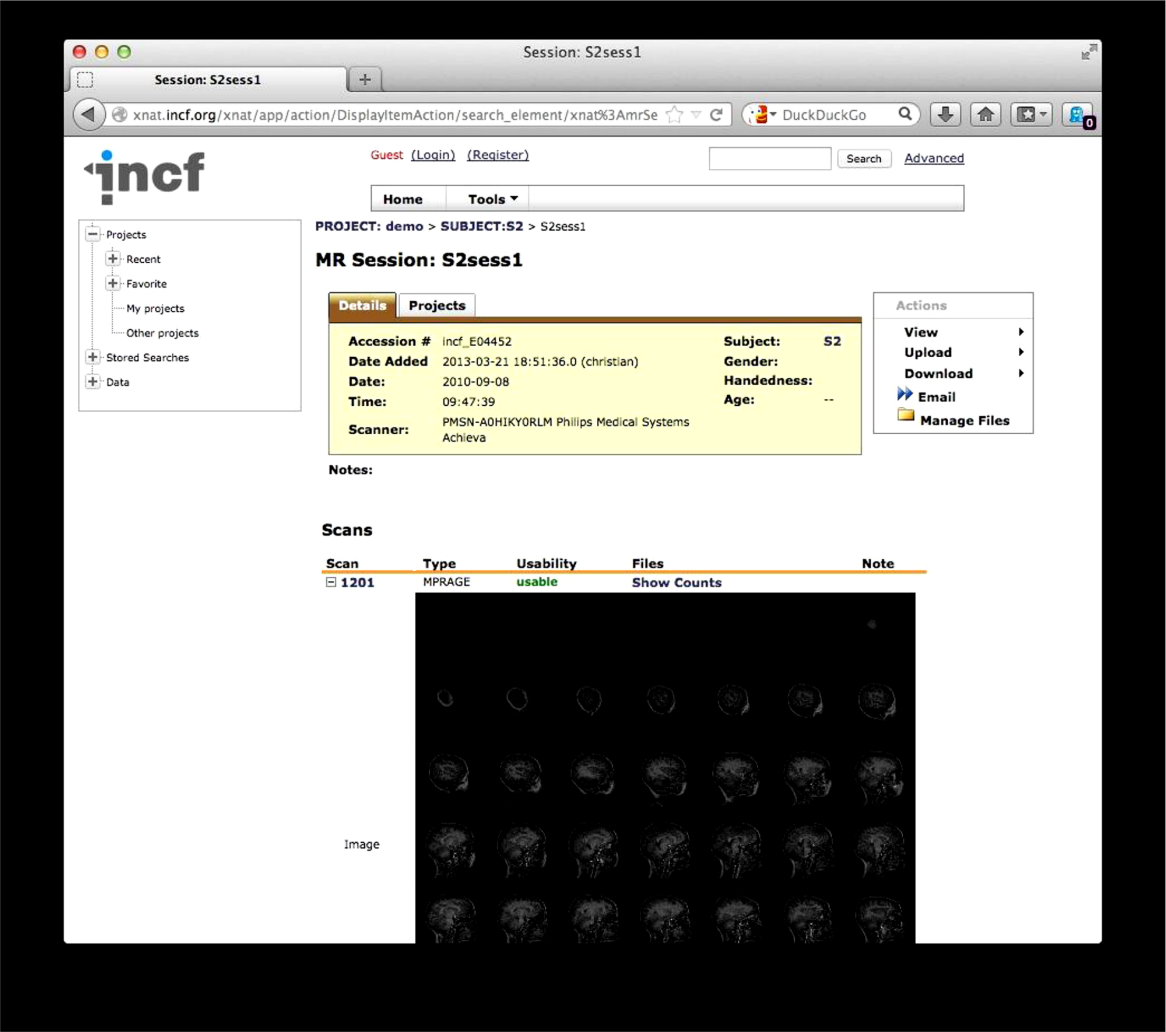
**The existing one-click XNAT archive.** Data is structured by project, subject, session, and scan. An automatically generated thumbnail image is also shown.

The archiving itself is a standard, built-in function of XNAT, which arranges the data into projects, subjects, sessions, and scans, after which thumbnail images are created for each scan (**Figure 6**). At this point the data is available for download, and users can browse or search the archive for data. After archiving, QA is launched.

#### Quality assessment

Once the data has been validated, QA runs are launched. QA procedures differ for various scan types, and the results are stored on the archive and sent to the user by e-mail. Currently, three types of QA are available for these scan types: structural, time series, and diffusion. Structural QA is run on any scan of type MPRAGE. Time series and diffusion QA is launched for every scan and allowed to fail if the data does not satisfy the prerequisites for these types (i.e., data with only one time point will file the time series QA, and data without diffusion gradient direction descriptions will fail the diffusion QA). QA begins by converting each scan to NIfTI-1 and NRRD, and the bundling the data and descriptors into an XCEDE-formatted file (Gadde et al., 2012). XCEDE-formatted data is required by the QA procedures. Even if the QA fails, these alternate data formats will be available for download on the archive.

***Structural QA.*** The structural QA is a custom procedure created for this system. This procedure calculates image intensity statistics over white matter, gray matter, CSF, whole brain, and the region exterior to the head. The signal to noise ratio (SNR) is defined as the mean image intensity in the brain divided by the standard deviation of the image intensity external to the brain.

FSL^25^ (Zhang et al., 2001; Smith, 2002; Smith et al., 2004; Jenkinson et al., 2005) is used to classify regions in the volume and calculate statistics, specifically:

• Brain and head are determined using bet image -A -m.
• Tissue types are determined using fast -t 1 image_brain, where image_brain is an output of bet.
• Statistics are calculated using fslstats, using -k to mask each region, -R for the minimum and maximum intensities, -r for the robust minimum and maximum intensities, -m for the mean intensity, -s for the intensity standard deviation, -v for the number of voxels and the volume.

As this is a new and custom structural image QA procedure designed as a simple proof of concept for this tool, it is imperfect and likely to evolve as it is used as we study the results obtained on large numbers of scans.

***Time series QA.*** Time series QA is performed by fmriqa_generate.pl, part of the BXH/XCEDE Tools suite^26^ (Friedman et al., 2006). This program takes XCEDE wrapped data and produces a web page reporting the results, including several plots. Examples of measures are the mean volume intensity at each time point and the center of mass (*x*, *y*, and *z*) at each time point. Plots of these measures can indicate at a glance if there is a variation at a given time point that warrants further investigation. The mean SNR and mean signal to fluctuation noise ratio (SFNR) are also calculated as part of this process.

***Diffusion QA.*** Diffusion QA is provided by DTIPrep^27^ (Liu et al., 2010) with default parameters (DTIPrep -w scan.nrrd -p default -d -c). The DTIPrep produces an XML report containing a number of pass/fail checks of basic image parameters (spatial information, basic gradient checks) followed by informational reports of other parameters (e.g., gradient directions) that can be examined for errors or possible problems. DTIPrep will also generate warnings of certain non-standard conditions that might warrant additional investigation (e.g., a non-standard number of gradient directions or suspicious b-values).

**FIGURE 7:**
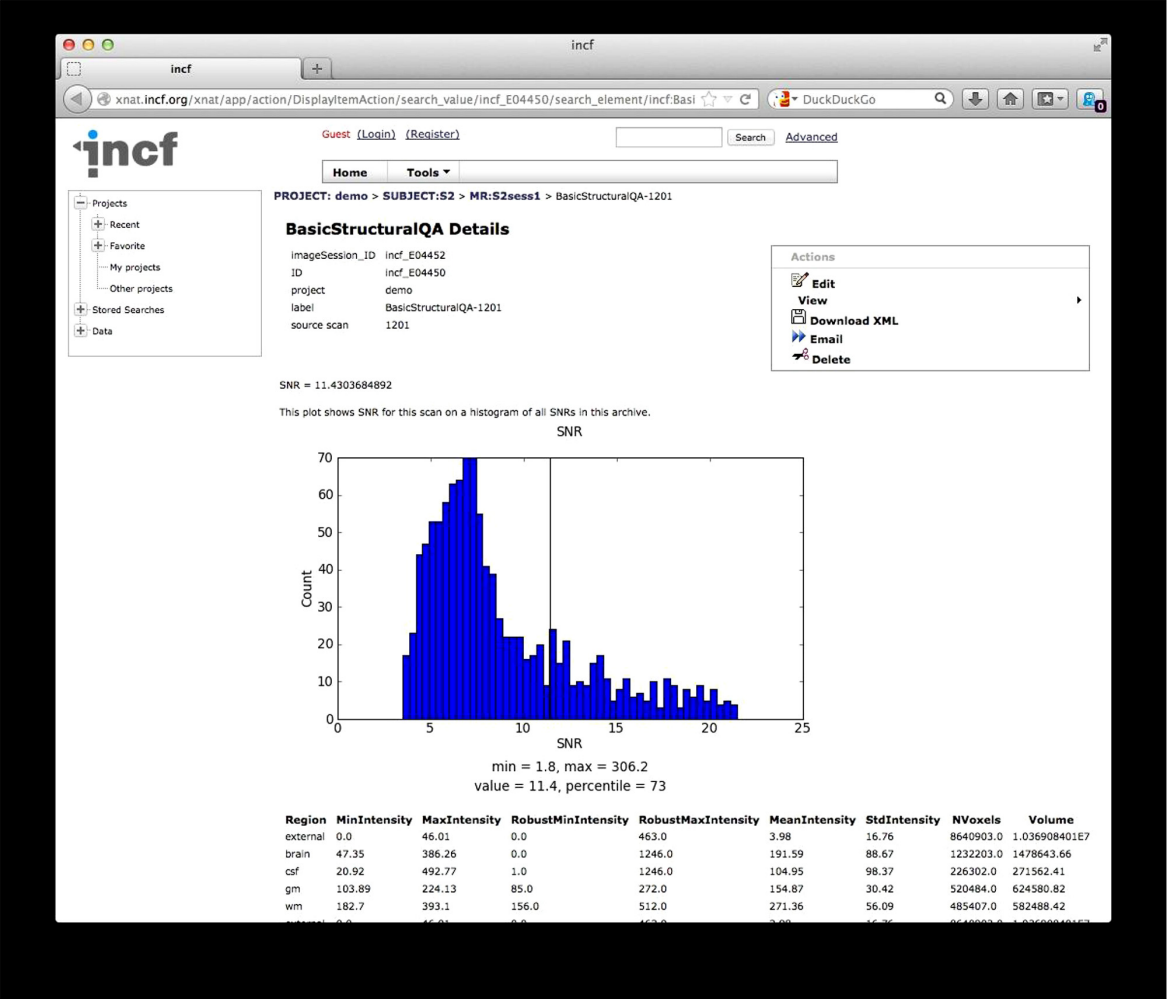
**Quality Assessment (QA) results.** The results from the structural QA are shown as a custom XNAT assessment. The SNR is plotted against a histogram of SNR values for a base set of data and for data in the archive. Raw values from the structural QA are shown at the bottom. Similar reports are created for time series QA and diffusion QA.

***QA reporting.*** Quality assessment results are parsed and stored on the archive as assessments, custom XNAT data types that allow for storage, management, and display of arbitrary data types. These assessments are accessible from the web front-end and are associated with the raw data for each scan (**Figure 7**).

While the diffusion QA is mainly informational with some pass/fail results, the SNR and SFNR calculated for the structural and time series QA procedures provide quantitative values that may not have much meaning in isolation but can be compared against other scans or collections of scans. For these QA reports, histograms of SNR and SFNR for similar scans in the database as well as for data in the 1000 Functional Connectomes^28^, as a reference dataset, are generated on the fly to give context of the SNR and SFNR values for these scans.

The archive web front-end presents other data as is, such as the raw values of tissue volume and voxel intensity statistics for each tissue type (structural QA), the intensity and motion plots (time series QA), and the diffusion pass/fail checks and gradient information (diffusion QA).

When this processing is complete, the user is notified by e-mail and given pointers to the data and to the QA results.

#### Data sharing

The data itself and the QA results are archived in a structured way and made publicly available in several formats. The user retains full control of the data, however, and can make the data private (on a project-by-project basis, following the XNAT security model) or can remove the data from the archive completely.

## DISCUSSION

The system was conceived to remove some of the technical barriers to data sharing and address some common excuses such as “it’s too hard,” “it takes too long,” “there’s nowhere that will publicly host my data,” and “I need to make sure the data is anonymized.” At this point, the system addresses all of these issues. With this basic functionality in place, the system can support other missions as well. There has been interest in this platform to support the NIH data sharing mandate and journals’ data sharing requirements. There has also been independent interest in QA measures and interest in the system providing further basic data analysis such as FreeSurfer^29^ (Dale et al., 1999; Fischl et al., 1999) reconstructions as a matter of course.

### LIMITATIONS

There are various limitations to the current system, on the user side, on the repository side, and on the system as a whole.

At this point, the user tools trade customizability for ease of use, but this does not have to be a strict tradeoff. Anonymization should be flexible, and the target of the upload should be customizable (allowing for multiple archives; this could mean archives with other processing on the back end, or local archives). With sensible defaults in place, adding these options does not need to stand in the way of basic usability. The tools do require user attention, but could be even more useful if a non-interactive mode were provided. The command line script could then be embedded in processing or other pipelines so data can be uploaded to an archive as part of the same mechanism that moves it from the scanner to a local lab for analysis, or to use an archive to do some initial analysis. The user tools are also limited as to what platforms they will run on (for the GUI tools), and the command-line script has several dependencies that must be installed by hand if the Debian package is not used. A web-based option for the user tool would be the ideal solution here, but would require that anonymization be performed on the server side or using local JavaScript code.

On the server side, we identify scans for structural QA by their declared scan types (MPRAGE). This could be extended by using a lexicon of scan types (MPRAGE, SPGR, FSPGR, etc) but this solution will not scale: much structural data will be always described in terms unfamiliar to the system, and the lexicon will be forever chasing data found in the real world. A better way of identifying scan types is likely to by inspection of scan parameters reported in DICOM fields combined with a lexicon of scan types. Allowing the user to specify the scan type unambiguously would also solve this problem.

One limitation of the system as a whole is its requirement for DICOM data. While the DICOM transfer protocol was useful for this initial prototype, other data formats (NIfTI-1, MGH, MINC, etc.) are more prevalent in day-to-day use within individual laboratories, and there is currently no good way to convert these files back to DICOM to prepare it for upload. Most imaging data starts as DICOM at the scanner, however, so this limitation is less of a problem as investigators begin to consider centralized archival of their data immediately upon acquisition. The restriction to DICOM data also limits the system to imaging data, while other modalities (e.g., EEG) are excluded from using the system.

Finally, the utility of the structural QA technique is currently unknown. We hope that as this is applied to more data, it will become clear how to interpret it and how to improve it. While the time series and diffusion QA procedures have been formalized more completely, it still remains to be seen exactly how to incorporate these metrics into practical implementations that indicate QA limits for data as a function of a desired use.

## CONCLUSION

What was conceived during a discussion of data sharing as a system to aid data sharing has now been implemented, providing users with a way to share data that addresses ease of use, anonymization, and storage and archiving, and even providing some basic processing results. The basic functionality is in place; users need only to start using the system. The fact that they haven’t is not a failure of the system; rather, it is a form of progress in ongoing data sharing efforts.

Providing this system that functions to its technical specifications has removed certain technical barriers, throwing into relief some of the social issues standing in the way of effective data sharing. Exposing these issues will allow us to better understand and focus on them. With “we can’t share” out of the way, we can better attack “we won’t share.” Data sharing has not been solved, but the discussion has been moved forward. And as further barriers are removed, we have in place an infrastructure for sharing and archiving.

## Conflict of Interest Statement

The authors declare that the research was conducted in the absence of any commercial or financial relationships that could be construed as a potential conflict of interest.

## Footnotes

1 http://grants.nih.gov/grants/policy/data_sharing

2 http://cancerimagingarchive.net/

3 http://ndar.nih.gov/

4 http://coins.mrn.org/

5 http://www.nitrc.org/projects/hid/

6 https://www.nitrc.org/projects/loris/

7 http://nidb.sourceforge.net/

8 http://www.xnat.org

9 https://wiki.xnat.org/display/XNAT/XNAT+Desktop

10 http://nrg.wustl.edu/software/dicom-browser/

11 http://code.google.com/p/pydicom/

12 http://code.google.com/p/httplib2/

13 http://dicom.offis.de/dcmtk.php.en

14 http://xnat.incf.org/

15 http://neuro.debian.net/

16 http://www.riverbankcomputing.com/software/pyqt/intro

17 http://github.com/incf/one_click

18 http://www.incf.org/

19 Representational state transfer, an architectural standard for communication between components in a distributed system.

20 ftp://medical.nema.org/medical/dicom/final/sup55_ft.pdf

21 https://wiki.nci.nih.gov/display/Imaging/Clinical+Imaging+Data+Sharing

22 http://nrg.wustl.edu/software/dicom-browser/

23 http://medical.nema.org/

24 http://www.nitrc.org/projects/xnat_extras/

25 http://www.fmrib.ox.ac.uk/fsl/

26 http://www.nitrc.org/projects/bxh_xcede_tools

27 http://www.nitrc.org/projects/dtiprep

28 http://www.nitrc.org/projects/fcon_1000

29 http://freesurfer.net/

## References

[B1] Biswal B. B. Mennes M. Zuo X. N. Gohel S. Kelly C. Smith S. M. (2010). Toward discovery science of human brain function. *Proc. Natl. Acad. Sci. U.S.A.* 107 4734–4739 10.1073/pnas.0911855107 20176931 PMC2842060

[B2] Book G. A. Anderson B. M. Stevens M. C. Glahn D. C. Assaf M. Pearlson G. D. (2013). Neuroinformatics Database (NiDB) – a modular, portable database for the storage, analysis, and sharing of neuroimaging data. *Neuroinformatics* 11 495–505 10.1007/s12021-013-9194-1 23912507 PMC3864015

[B3] Dale A. M. Fischl B. Sereno M. I. (1999). Cortical surface-based analysis. I. Segmentation and surface reconstruction. *Neuroimage* 9 179–194 10.1006/nimg.1998.0395 9931268

[B4] Das S. Zijdenbos A. P. Harlap J. Vins D. Evans A. C. (2012). LORIS: a web-based data management system for multi-center studies. *Front. Neuroinform.* 5:37 10.3389/fninf.2011.00037 PMC3262165 22319489

[B5] De Schutter E. (2010). Data publishing and scientific journals: the future of the scientific paper in a world of shared data. *Neuroinformatics* 8 151–153 10.1007/s12021-010-9084-8 20835853

[B6] Di Martino A. Yan C. G. Li Q. Denio E. Castellanos F. X. Alaerts K. (2013). The autism brain imaging data exchange: towards a large-scale evaluation of the intrinsic brain architecture in autism. *Mol. Psychiatry* 10.1038/mp.2013.78 [Epub ahead of print]. PMC4162310 23774715

[B7] Fischl B. Sereno M. I. Dale A. M. (1999). Cortical surface-based analysis. II: inflation, flattening, and a surface-based coordinate system. *Neuroimage* 9 195–207 10.1006/nimg.1998.0396 9931269

[B8] Friedman L. Glover G. H Fbirn Consortium. (2006). Reducing interscanner variability of activation in a multicenter fMRI study: controlling for signal-to-fluctuation-noise-ratio (SFNR) differences. *Neuroimage* 33 471–481 10.1016/j.neuroimage.2006.07.012 16952468

[B9] Gadde S. Aucoin N. Grethe J. S. Keator D. B. Marcus D. S. Pieper S. (2012). XCEDE: an extensible schema for biomedical data. *Neuroinformatics* 10 19–32 10.1007/s12021-011-9119-9 21479735 PMC3836560

[B10] Halchenko Y. O. Hanke M. (2012). Open is not enough. Let’s take the next step: an integrated community driven computing platform for neuroscience. *Front. Neuroinform.* 6:22 10.3389/fninf.2012.00022 PMC3458431 23055966

[B11] Hall D. Huerta M. F. McAuliffe M. J. Farber G. K. (2012). Sharing heterogeneous data: the national database for autism research. *Neuroinformatics* 10 331–339 10.1007/s12021-012-9151-4 22622767 PMC4219200

[B12] Jenkinson M. Pechaud M. Smith S. (2005). “BET2: MR-based estimation of brain, skull and scalp surfaces,” in *Proceedings of the Eleventh Annual Meeting of the Organization for Human Brain Mapping* Oxford, UK

[B13] Liu Z. Want Y. Gerig G. Gouttard S. Tao R. Fletcher T. (2010). Quality control of diffusion weighted images. *Proc. Soc. Photo Opt. Instrum. Eng.* 7628 10.1117/12.844748 PMC3864968 24353379

[B14] Marcus D. S. Olsen T. R. Ramaratnam M. Buckner R. L. (2007). The extensible neuroimaging archive toolkit: an informatics platform for managing, exploring, and sharing neuroimaging data. *Neuroinformatics* 5 11–34 10.1385/NI:5:1:11 17426351

[B15] Ozyurt I. B. Keator D. B. Wei D. Fennema-Notestine C. Pease K. R. Bockholt J. (2010). Federated web-accessible clinical data management within an extensible neuroimaging database. *Neuroinformatics* 8 231–249 10.1007/s12021-010-9078-6 20567938 PMC2974931

[B16] Poline J. B. Breeze J. L. Ghosh S. Gorgolewski K. Halchenko Y. O. Hanke M. (2012). Data sharing in neuroimaging research. *Front. Neuroinform.* 6:9 10.3389/fninf.2012.00009 PMC3319918 22493576

[B17] Prior F. W. Clark K. Commean P. Freymann J. Jaffe C. Kirby J. (2013). TCIA: an information resource to enable open science. *Conf. Proc. IEEE Eng. Med. Biol. Soc.* 2013 1282–1285 10.1109/EMBC.2013.6609742 24109929 PMC4257783

[B18] Scott A. Courtney W. Wood D. de la Garza R. Lane S. King M. (2011). COINS: an innovative informatics and neuroimaging tool suite built for large heterogeneous datasets. *Front. Neuroinform.* 5:33 10.3389/fninf.2011.00033 PMC3250631 22275896

[B19] Smith S. M. (2002). Fast robust automated brain extraction. *Hum. Brain Mapp.* 17 143–155 10.1002/hbm.10062 12391568 PMC6871816

[B20] Smith S. M. Jenkinson M. Woolrich M. W. Beckmann C. F. Behrens T. E. J. Johansen-Berg H. (2004). Advances in functional and structural MR image analysis and implementation as FSL. *NeuroImage* 23 208–219 10.1016/j.neuroimage.2004.07.051 15501092

[B21] Zhang Y. Brady M. Smith S. (2001). Segmentation of brain MR images through a hidden Markov random field model and the expectation-maximization algorithm. *IEEE Trans. Med. Imaging* 20 45–57 11293691 10.1109/42.906424

